# The T Stage of Papillary Thyroid Cancer and Its Effect on Regional Complications of Thyroidectomy

**DOI:** 10.1002/oto2.70213

**Published:** 2026-08-17

**Authors:** Fahad Rind, Songzhu Zhao, Stephen Kang, Catherine T. Haring, Amit Agrawal, Enver Ozer, Matthew O. Old, Ricardo L. Carrau, Nolan B. Seim

**Affiliations:** ^1^ Division of Head and Neck Oncology Ohio State University Comprehensive Cancer Center, Arthur G. James Cancer Hospital and Richard J. Solove Research Institute Columbus Ohio USA; ^2^ Department of Otolaryngology–Head and Neck Surgery The Ohio State University Wexner Medical Center Columbus Ohio USA

**Keywords:** papillary thyroid cancer, surgery, thyroid cancer, T stage

## Abstract

**Objectives:**

To evaluate the impact of increasing AJCC T stage on postoperative morbidity in patients undergoing thyroidectomy for papillary thyroid cancer.

**Study Design:**

Retrospective cross‐sectional database study.

**Setting:**

The American College of Surgeons National Surgical Quality Improvement Program (NSQIP), which collects surgical outcomes data from ~700 US hospitals.

**Methods:**

NSQIP Participant User Files were queried for thyroidectomy procedures (CPT codes 60210, 60212, 60220, 60225, 60240, 60252, 60254, 60260) from 2016 to 2020 with a pathology‐confirmed diagnosis of papillary thyroid cancer. Primary exposure was T stage and outcomes included hoarseness, hypocalcemia, and hematoma formation. Multivariate analysis controlled for N stage, total thyroidectomy, multifocality, and neck dissection.

**Results:**

A total of 10,901 cases were identified (74.2% female; mean age 50.1 ± 15.1 years). Compared to early‐stage tumors, T3 cancers were associated with increased risk of hoarseness (OR 1.614, 95% CI 1.260‐2.074, *P* < .01) and 30‐day hypocalcemia (OR 1.296, 95% CI 1.022‐1.645, *P* = .03). Upstaging to T4 further increased risks of hoarseness (OR 2.971, 95% CI 2.039‐4.282, *P* < .01), hematoma (OR 2.266, 95% CI 1.027‐4.999, *P* = .04), and hypocalcemia (OR 1.542, 95% CI 1.007‐2.364, *P* < .05). No significant morbidity difference was observed between T1 and T2 tumors.

**Conclusion:**

Increasing T stage is associated with significantly higher postoperative morbidity in patients with papillary thyroid cancer, particularly when advancing to T3 or T4. These findings highlight the importance of preoperative risk assessment and surgical planning in advanced disease.

Thyroid surgery has been performed for centuries with the first partial thyroidectomy ever recorded in 1791 by Pierre Joseph Desault.^1^ Over the past 200 years, the procedure has been studied and written on in detail^2^ with present‐day advances in sterility,^3^, ^4^ hemostasis,^5^ and surgical technique^5^ having taken it from a controversial undertaking to a low‐risk outpatient procedure.^1^ The thyroid gland itself is highly vascularized and sits within millimeters of the parathyroid glands,^6^ thyroid vasculature,^7^ trachea, esophagus, and branches of the vagus nerves.^8^

The possibility of operative complication related to this nearby anatomy can precipitate morbidity in the form of vocal cord paresis/paralysis (0.9%‐1.1%),^9^, ^10^ hematoma (0.7%‐5.4%),^9^, ^10^, ^11^ calcium dysregulation (1.9%‐27%),^9^, ^12^, ^13^, ^14^, ^15^ dysphagia^16^ and impaired cosmesis^17^ secondary to thyroidectomy procedures. A meta‐analysis showed the rates of recurrent laryngeal nerve injury to range from 0% to 38.4% and aggregated these rates to a 9.3% incidence of temporary RLN palsy and 2.3% incidence of permanent RLN palsy.^18^

The TNM staging of thyroid cancer correlates with overall survival.^19^ The T (Tumor) portion of the staging is a measure of a tumor's size, extension, and local invasion. Thus, it can be the major indicator of local structures affected or invaded by the tumor and the intricate anatomy the surgeon may have to navigate and preserve. However, the effect of T stage on local head and neck morbidity is not well studied. The significance of T stage's effect on morbidity would help in the risk stratification and prognostication of surgery. This data could potentially inform a cost‐benefit analysis when undertaking surgery and lend caution toward more frequent complications. The National Surgical Quality Improvement Program (NSQIP) database allows for national‐level analysis of surgical outcomes of specific, common procedures like thyroidectomy. A recent review of the database showed hoarseness to be independently associated with total thyroidectomy, thyroid malignancy, and age >65 years.^20^ This study aims to determine the risk of head and neck complications when operating on thyroid cancers of different T stages.

## Methodology

The 2016 to 2020 ACS‐NSQIP Targeted Procedure Thyroidectomy Participant Use Files (PUF) contain thyroid cancer‐specific fields allowing for the staging of tumors, and type of cancer. The NSQIP is a quality improvement database built from a random unbiased sample of surgical cases conducted at participating sites across the United States. Ethical board review requirements were waived due to de‐identified handling of publicly available observational data in the NSQIP. Inclusion criteria consisted of cases with principal current procedure terminology (CPT) codes 60210, 60212, 60220, 60225, 60240, 60252, 60254, or 60260 were used to identify surgery on papillary thyroid cancer cases in patients of at least 18 years of age. Exclusion criteria included CPT codes for retrosternal thyroid disease (60271) due to incomparable intraoperative complexity. These records were merged with the corresponding case ID Numbers from the ACS NSQIP PUF for access to general demographics and surgical outcomes. The primary outcomes of interest were hoarseness, hematoma formation, hypocalcemia, and hypocalcemic events (defined as urgent medical evaluation, readmission, or IV supplementation of calcium due to hypocalcemia) and were detected in the 30 days postoperative period. Descriptive analysis was conducted on the patient demographics, comorbidities, operative factors, and postoperative complication rates. The T stage on surgical pathology was grouped into T1, T2, T3, and T4 to allow comparisons between T stages.

Logistic regressions were employed to identify the comparative risk of odds of different disease states and statistical analysis was conducted using *R 4.2*. *P *< .05 were considered statistically significant. The risk imparted by identified risk factors were recorded as odds ratios (OR) with 95% confidence intervals (CI) and *P*‐values. In cases where data was not available or labeled unknown, only known values entered logistic regression models. Multivariate logistic regressions controlled for the N stage of the neck, total thyroidectomy procedure, multifocality of disease, and accompanying neck dissection procedure. Multivariate analysis of the risk of neck hematoma additionally assessed the use of a neck drain.

## Results

### Baseline Subject Characteristics (see Table 1)

**Table 1:** Descriptive Data of All Included Cases Capturing Their Baseline Demographic, Comorbidity Status, and Postoperative Course

Demographics	Sex (female)	8084 (74.2%)
	Race	White: 5536 (50.8%)
		Unknown: 3808 (34.9%)
		Asian: 846 (7.8%)
		Black: 617 (5.7%)
		Other: 94 (0.8%)^a^
	Age	Mean: 50.1 year
		Standard deviation: 15.1 years
Operative factors	Inpatient outpatient	Outpatient: 5841 (53.6%)
		Inpatient: 5060 (46.4%)
	Wound classification	Class 1—Clean: 10651 (97.7%)
		Class 2—Clean/Contaminated: 231 (2.1%)
		Class 3—Contaminated: 18 (.2%)
		Class 4—Dirty/Infected: 1 (<0.1%)
	ASA Classification of Surgery	Class 2—Mild Disturbances: 6168 (56.6%)
		Class 3—Severe Disturbances: 3653 (33.5%)
		Class 1—No disturbances: 825 (7.6%)
		Class 4—Life‐threatening: 191 (1.8%)
		Class 5—Moribund 0 (0%)
	Elective surgery	Elective: 10,780 (98.9%)
Comorbid/Acute health	Diabetic	Yes: 1421 (13.0%)
	Smoker in the last year	Smoker: 1165 (10.7%)
	Dyspnea	No dyspnea: 10,381 (95.2%)
		Upon moderate exertion: 500 (4.6%)
		At rest: 20 (0.2%)
		ie, Yes: 520 (4.8%)
	Bleeding disorders	88 (0.8%)
	Ventilator dependence	6 (0.1%)
	COPD	147 (1.3%)
	CHF	23 (0.2%)
	Dialysis dependent	46 (0.4%)
	Disseminated cancer	193 (1.8%)
	Steroid use	290 (2.7%)
	Weight loss	38 (0.3%)
	Pneumonia	30 (0.3%)
	Reintubation	33 (0.3%)
	Pulmonary embolism	11 (0.1%)
	UTI	32 (0.3%)
	Return to OR	141 (1.3%)
	Neck dissection	5025 (39.9%)
	Harmonic scalpel use	6148 (58.7%)
	T stage	T1: 6334 (58.1%) cases (3518 (32.3%) T1a and 2815 (25.8%) T1b)
		T2: 2129 (19.5%) cases
		T3: 1831 (14.7%) cases, ie, 143 (1.3%) T3a and 87 (0.8%) T3b
		T4: 191 (1.8%), ie, 165 T4a (1.5%), 12 T4b (0.1%)
	Nodes	Nx: 3508 cases (32.2%)
		N0: 3822 cases (35.1%)
		N+: 3183 cases (29.2%)
		N1: 337 cases (3.1%)
		N1a: 1707 cases (15.7%)
		N1b: 1139 cases (10.4%)
	Hypocalcemia	No: 10156 (93.2%)
		Yes: 612 (4.0%)
	Hypocalcemia (over the next 30 days)	No: 9826 (92.4%)
		Yes: 805 (7.6%)
	Hypocalcemic events	491 (4.5%)
	Hoarseness	719 (6.6%)
	Hematoma	Yes: 170 (1.6%)

^a^ Other races included 67 American Indian/Alaskan Natives, 26 Native Hawaiian or other pacific islanders and one nonspecified race.

Our query of the ACS NSQIP PUFs yielded 10,901 cases of oncologic thyroidectomies for the treatment of papillary thyroid cancer. The majority of cases concerned female patients (n = 8084, 74.2%) with a mean age of 50.1 years (standard deviation 15.1 years). Most surgeries were conducted in an outpatient setting (n = 5841, 53.6%). Operations were most commonly Class 1 wounds (Clean; n = 10,651, n = 97.7%) and most commonly assessed to be an ASA class 2 surgery (n = 6168, 56.6%). Patients were overall healthy with low rates of diabetes (n = 1421, 13.0%), smoking (n = 1165, 10.7%), dyspnea (n = 520, 4.8%), bleeding disorders (n = 88, 0.8%), COPD (n = 147, 1.3%), CHF (n = 23, 0.3%), dialysis dependence (n = 46, 0.4%), disseminated cancer (n = 193, 1.8%), weight loss (38, 0.3%), use of immunosuppressants (n = 290, 2.7%), and only 396 patients presented with evidence of thyrotoxicity (4.7%).

Postoperative courses were minimally complicated with low rates of dehiscence (n = 5), postoperative reintubation (n = 33), pulmonary embolism (n = 11), urinary tract infections (n = 32), and stroke (n = 3) and patients spent on average 1.24 days admitted after surgery. In total, 141 cases were required to return to OR (1.3%).

### Head and Neck‐Specific Complications

#### Postoperative Hoarseness

The overall rate of hoarseness after surgery was 6.6%, however, as T stage increased from T1 to T4 the rate of hoarseness increased exponentially (5.5%‐5.9% to 9.6%‐26.7%; see Table 2). For low‐stage disease, increased from T1 to T2 did not significantly increase the risk of postoperative hoarseness (*P* = .813). On multivariate analysis, upstaging from T1 to T3 increased the risk of hoarseness by 67% (OR 1.559, 95% CI 1.364‐2.036, *P* < .001), and upstaging from T1 to T4 disease increased the risk of hoarseness (5.5%‐26.7%; OR 5.842, 95% CI 4.028‐8.382, *P* < .001; see Table 3). When comparing with T2 disease, advanced‐stage primary tumors, T3 cancer and T4 cancers both conferred a significant increase in the risk of hoarseness. Similarly, upstaging from T3 to T4 disease almost tripled the risk of postoperative hoarseness on multivariate analysis (OR 2.971, 95% CI 2.039‐4.282, *P* < .001).

**Table 2:** Descriptive Data Table Showing the Distribution of Complications as Divided by the T Stage

	T1	T2	T3	T4	All
Hoarseness	369/6696	124/2111	175/1820	51/191	719/10,818
(comp. rate)	5.5%	5.9%	9.6%	26.7%	6.6%
Neck hematoma	98/6562	28/2062	31/1781	10/186	167/10,424
(comp. rate)	1.5%	1.3%	1.7%	5.4%	1.6%
Hypocalcemia	325/6672	118/2092	138/1814	31/190	612/10,156
(comp. rate)	5.3%	5.6%	7.6%	16.3%	6.0%
30 d Hypocal.	445/6590	148/2070	180/1785	32/186	805/10,631
(comp. rate)	6.7%	7.1%	10.1%	17.2%	7.6%
Hypocal. Events	264/6752	93/2129	112/1829	22/191	491/10,901
(comp. rate)	3.9%	4.4%	6.1%	11.5%	4.5%

**Table 3:** Logistic Regression Analysis Assessing the Risk of Head and Neck Complications in the Included Dataset

		Univariate risk of complication		Multivariate risk of complication	
T stage comparator	vs	OR	*P*‐value	OR	*P*‐value
Hoarseness*					
T1	T2	1.070 (0.865, 1.316)	.527	1.027 (0.822, 1.274)	.813
	T3	**1.824 (1.509, 2.198)**	**<.001**	**1.669 (1.364, 2.036)**	**<.001**
	T4	**6.246 (4.423, 8.696)**	**<.001**	**5.842 (4.028, 8.382)**	**<.001**
T2	T3	**1.705 (1.344, 2.169)**	**<.001**	**1.614 (1.260, 2.074)**	**<.001**
	T4	**5.837 (4.016, 8.401)**	**<.001**	**4.943 (3.309, 7329)**	**<.001**
T3	T4	**3.424 (2.382, 4.865)**	**<.001**	**2.971 (2.039, 4.282)**	**<.001**
Postoperative hematoma formation**					
T1	T2	0.896 (0.577, 1.347)	.609	0.897 (0.570, 1.365)	.623
	T3	1.230 (0.815, 1.810)	.307	1.151 (0.747, 1.731)	.510
	**T4**	**3.684 (1.777, 6.840)**	**<.001**	**3.251 (1.428, 6.682)**	**.002**
T2	T3	1.374 (0.828, 2.296)	.220	1.276 (0.756, 2.169)	.362
	**T4**	**4.114 (1.875, 8.336)**	**<.001**	**3.035 (1.230, 7.006)**	**.012**
**T3**	**T4**	**2.995 (1.380, 5.963)**	**.003**	**2.266 (1.027, 4.999)**	**.043**
Hypocalcemia (30‐day)*					
T1	T2	0.910 (0.742, 1.110)	.358	0.936 (0.762, 1.143)	.523
	T3	1.063 (0.874, 1.286)	.533	**1.243 (1.022, 1.506)**	**.028**
	T4	**2.869 (1.906, 4.192)**	**<.001**	**1.825 (1.190, 2.723)**	**.004**
T2	T3	**1.456 (1.161, 1.830)**	**.001**	**1.296 (1.022, 1.645)**	**.032**
	T4	**2.698 (1.756, 4.044)**	**<.001**	**1.793 (1.139, 2.761)**	**.010**
T3	T4	**1.853 (1.211, 2761)**	**<.001**	**1.542 (1.007, 2.364)**	**.046**
Hypocalcemic events (30 day)*					
T1	T2	1.123 (0.878, 1.424)	.348	1.016 (0.784, 1.306)	.903
	T3	**1.603 (1.273, 2.006)**	**<.001**	**1.338 (1.047, 1.700)**	**.019**
	T4	**3.199 (1.967, 4.966)**	**<.001**	**2.301 (1.379, 3.683)**	**<.001**
T2	**T3**	**1.428 (1.078, 1.897)**	**.013**	1.287 (0.959, 1.731)	.093
	**T4**	**2.850 (1.171, 4.573)**	**<.001**	**1.886 (1.100, 3.123)**	**.017**
T3	**T4**	**1.996 (1.203, 3.178)**	**.005**	1.599 (0.950, 2.587)	.065

Emboldened variables signify a statistical significance of association was found between variables.

Abbreviation: OR, odds ratio.

*Multivariate analysis controlled for N stage, multifocality of disease, total thyroidectomy procedure, and performance of neck dissection.

**Multivariate analysis of neck hematoma formation controlled for one additional covariate, drain placement.

#### Postoperative Hematoma

The aggregate rate of postoperative hematoma was 1.6% across the dataset (see Table 2) and increased inconsistently as the T stage was increased, that is, 1.5%‐1.3% to 1.7%‐5.4%. T4 disease was the only T‐stage predictor that significantly increased the risk of postoperative hematoma in this dataset. In multivariate analysis, upstaging to T4 from T1 (OR 3.251, 95% CI 1.428‐6.682, *P *= .002), T2 (OR 3.035, 95% CI 1.230‐7.006, *P* = .012), and T3 (OR 2.266, 95% CI 1.027‐4.999, *P* = .043) all significantly increased the risk of postoperative hematoma when controlling for drain use.

#### 30‐Day Hypocalcemia

The overall rate of hypocalcemia after thyroidectomy was 7.6% for the data set and this increased exponentially with increasing T stage (6.7%‐7.1% to 10.1%‐17.2%; see Table 2).

When comparing to T1 cases, T3 (OR 1.243, 95% CI 1.022‐1.506, *P* = .028) and T4 (OR 1.825, 95% CI 1.190‐2.723, *P* = .004) tumors showed a significant increase in the risk of 30‐day hypocalcemia. Upstaging from T2 to T3 (OR 1.296. 95% CI 1.022‐1.645, *P* = .032) and T4 (OR 1.793, 95% CI 1.139‐2.761, *P* = .010) disease increased the risk of hypocalcemia on multivariate analysis. The effect of T4 stage increasing the risk of 30‐day hypocalcemia persisted when upstaging from T3 (OR 1.542, 95% CI 1.007‐2.364, *P* = .046).

#### Hypocalcemic Events (30‐Days)

The overall rate of hypocalcemic events was 4.5% and the rates increased exponentially with increasing T stage (3.9%‐4.4% to 6.1%‐11.5%). In the multivariate analysis, upstaging from T1 to T3 (OR 1.338, 95% CI 1.047‐1.700, *P* = .019) and T1 to T4 (OR 2.301, 95% CI 1.379‐3.683, *P* < .001) conferred significant increases in the risk of hypocalcemic events after thyroidectomy. Upstaging from T2 to T3 did not have a significant effect, however, upstaging from T2 to T4 increased the risk by 89% (OR 1.886, 95% CI 1.100‐3.123, *P* = .017) on multivariate analysis. However, when comparing advanced stage tumors, T3 to T4 did not significantly increase the risk of hypocalcemic events (*P* = .065).

## Discussion

The ACS NSQIP allows access to large datasets that far outstretch the possible clinical experience of a single center's lifetime. This allows the establishment of high‐powered correlations between risk factors and complication rates. The thyroidectomy‐specific PUF additionally allows the mapping of specific head and neck complications causing significant social and functional morbidity in relatively healthy patients undergoing this surgical treatment. This study aimed to utilize the massive sample sizes available from surgical outcomes data collected across over 700 sites to investigate the risk of head and neck complications in different T stages of thyroid cancer to improve pre‐operative counseling and focus anticipated expectations.

**Figure 1:**
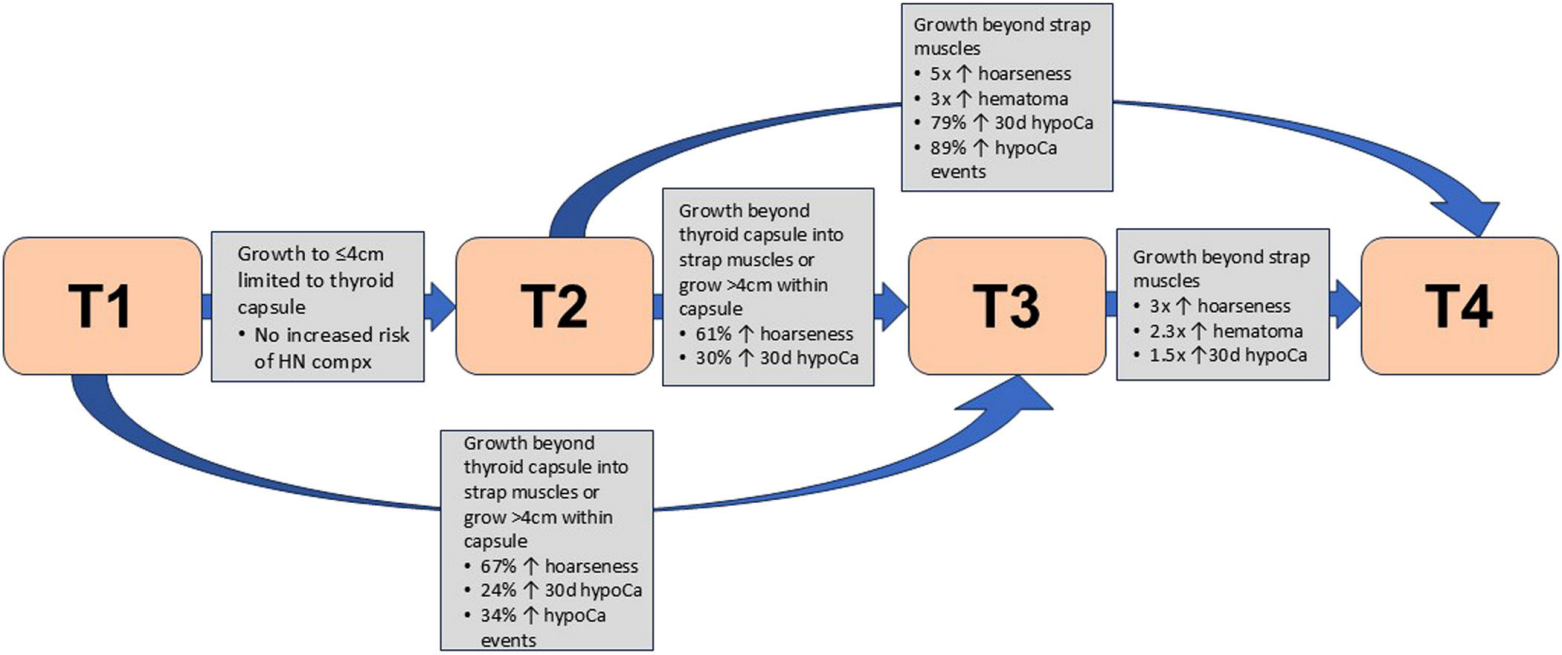
Flowchart illustrating the escalating risk of morbidity with the extent of tumor growth and local invasion. 30d, 30 day; compx, complications; HN, head and neck; hypoCa, hypocalcemia.

There was no significant increase in the risk of our chosen head and neck complications in lower‐stage disease, when staging increases from T1 to T2 (Figure 1). This shows that despite absolute differences in the rates of complication in this range, there is no significant increase in risk when treating intrathyroidal disease of less than 4 cm in its greatest dimension. Once staging increases from T2 to T3, however, there is a significantly greater risk of postoperative hoarseness and 30‐day hypocalcemia, highlighting the significance of thyroid cancer extending from within the thyroid gland to invading strap muscles or having size over 4 cm. Upstaging from T3 to T4 was associated with a greater risk of postoperative hoarseness, neck hematoma formation, and 30‐day hypocalcemia as expected as well. Of course, T4 tumors involve a wide spectrum of advanced locoregional disease that can include invasion into and through the strap muscles as well as other critical structures in the central neck including the larynx, trachea, esophagus, and/or subcutaneous tissues.

Postoperative hematoma formation was only correlated with T4 staging when controlled for drain use. The lack of drain use has been shown to reduce the need for intramuscular analgesia and shorten the length of stay.^21^, ^22^, ^23^ This paradigm shift is likely changing, however, with improved protocol such as patients pulling their own drains at home.^24^ The decision to place a drain is a surgeon's choice, therefore, though its purpose may be to minimize serosanguinous accumulations its presence may indicate an experience of surgical complexity by the surgeon and was thus controlled for in this study.

While never quantified in this large of a series, but as expected, there was a non‐linear increase in the magnitude of the associated risk of hypocalcemia, 30‐day hypocalcemia, hypocalcemic events, and hoarseness indicating a drastic increase in local morbidity in higher T‐stage thyroid cancers. The known significant risk factors of postthyroidectomy hoarseness included age, secondary operation, nodal metastasis, VC paralysis, T4a tumor stage, extra‐thyroidal extension, and trachea‐esophageal groove involvement.^17^, ^25^, ^26^, ^27^, ^28^, ^29^ Therefore, the covariates in our model were chosen with the same rationale.

An analysis of the 2016 data from this database was conducted by Barrows et al^30^ and found a significant increase in the rates of hypocalcemia, hoarseness, readmission, and medical complications. However, on multivariate analysis, the study found no association between the T stage and complications. Our study benefitted from multiple years of data resulting in a significantly larger sample size.

A similar analysis of the 2016 to 2018 data of thyroidectomy‐specific NSQIP concluded hoarseness occurred at a rate of 6% after thyroidectomy which is slightly more than the 5.7% found in our study.^31^ However, it isn't apparent that retrosternal thyroid cases were excluded from Kim et. al's publication, possibly explaining this slight difference. An association with tumor T stage was also not reported. Thyroidectomies in the hands of high volume surgeons have been shown to have less postoperative morbidity, shorter lengths of stay, and shorter anesthesia time.^32^ Our paper could suggest the clinical benefit of operative support from high‐volume surgeons when treating T3 and T4 PTC. The extension of neck dissections into level 6 and 7 were expected to have worse outcomes due to wider instrumentation of the anatomy, however, our logistic regression analysis found no such effect in all T‐stage groups (*P* > .05).

The association of regional complications with the T stage was anticipated as the classification correlates with increasing size and extent of local extension. The findings confirm the increase in morbidity in early‐stage vs advanced disease (T1 and 2 compared to T3 and T4) given the sequential jump in data's significance on T stage as a significant risk factor in hypocalcemia, 30‐day hypocalcemia, hypocalcemic events, and hoarseness. The need for central neck dissections (level 6) has been associated with an increased prevalence of hypocalcemia.^33^

This study represents the analysis of a very large dataset of cases from a wide variety of healthcare settings, surgical skill, and patient populations earning the conclusion's generalizability and robustness. It investigates a portion of the literature that has not been studied at the scale of this study and so may be of use in predicting. However, the study is limited by its retrospective and observational nature from which causation cannot be concluded. Additionally, several covariates could not be controlled for when using a national scale database such as the surgeon volume and experience that may help to inform an iatrogenic nature of complications recorded.

## Conclusion

This multivariate investigation into the effect of primary tumor staging in papillary thyroid cancer on head and neck complications showed there were significant increases in the risk of head and neck complications with increasing T stage. This study shows that on a multivariate level, the T stage of thyroid carcinoma is an independent risk factor for the postoperative development of 30‐day hypocalcemia, hypocalcemic events, and hoarseness of voice. In early‐stage disease, no increases in the risk of hematoma, hoarseness, hypocalcemia, and hypocalcemic events when comparing T1 and T2 cancers. This provides context in which to better counsel patients with papillary thyroid cancer on potential morbidity and recovery time following a very common oncologic surgery.

## Author Contributions

**Fahad Rind**, contributed significantly to project conception, analysis, writing and submission; **Songzhu Zhao**, contributed significantly to project conception, analysis, and writing; **Stephen Y. Kang**, contributed significantly to project conception, analysis, and writing; **Catherine T. Haring**, contributed significantly to project conception, analysis, and writing; **Amit Agrawal**, contributed significantly to project conception, analysis, and writing; **Enver Ozer**, contributed significantly to project conception, analysis, and writing; **Matthew O. Old**, contributed significantly to project conception, analysis, and writing; **Ricardo L. Carrau**, contributed significantly to project conception, analysis, and writing; **Nolan B. Seim**, contributed significantly to project conception, analysis, and writing, submission and direction.

## Disclosures

### Competing interests

None.

### Funding source

None.
